# Alternation of plasma fatty acids composition and desaturase activities in children with liver steatosis

**DOI:** 10.1371/journal.pone.0182277

**Published:** 2017-07-31

**Authors:** Man-Chin Hua, Hui-Min Su, Tsung-Chieh Yao, Ming-Ling Kuo, Ming-Wei Lai, Ming-Han Tsai, Jing-Long Huang

**Affiliations:** 1 Department of Pediatrics, Chang Gung Memorial Hospital, Keelung, Taiwan; 2 Chang Gung University College of Medicine, Taoyuan, Taiwan; 3 Department of Physiology, National Taiwan University College of Medicine, Taipei, Taiwan; 4 Division of Allergy, Asthma and Rheumatology, Department of Pediatrics, Chang Gung Memorial Hospital, Linkou Branch, Taoyuan, Taiwan; 5 Department of Microbiology and Immunology, Graduate Institute of Biomedical Sciences, College of Medicine, Chang Gung University, Taoyuan, Taiwan; 6 Division of Gastroenterology, Department of Pediatrics, Chang Gung Memorial Hospital, Linkou Branch, Taoyuan, Taiwan; 7 Liver research center, Chang Gung Memorial Hospital, Linkou Branch, Taoyuan, Taiwan; University of Illinois, UNITED STATES

## Abstract

**Objective:**

The aim of this study was to investigate changes in plasma fatty acids proportions and estimated desaturase activities for variable grading of liver steatosis in children.

**Methods:**

In total, 111 schoolchildren (aged 8–18 years) were included in the analysis from March 2015 to August 2016. Anthropometric evaluation, liver ultrasound examination and scoring for nonalcoholic fatty liver disease (NAFLD score = 0–6), and biochemical and plasma fatty acids analysis were performed. We compared the composition ratio of fatty acids between children with high-grade liver steatosis (NAFLD score = 4–6), low-grade liver steatosis (NAFLD score = 1–3), and healthy controls (NAFLD score = 0). In addition, correlation coefficients (r) between NAFLD score, metabolic variables, and estimated activity of desaturase indices (stearoyl-coenzyme A desaturase-1 (SCD1), delta-5 and delta-6 desaturase) were calculated.

**Results:**

Compared with healthy controls, children with liver steatosis showed a higher proportion of monounsaturated fatty acids (21.16 ± 2.81% vs. 19.68 ± 2.71%, *p* = 0.024). In addition, children with high- grade liver steatosis exhibited higher proportions of palmitic acid (C16:0), palmitoleic acid (C16:1n-7), dihomo-γ-linolenic acid (C20:3n-6), adrenic acid (C22:4n-6), and docosapentaenoic acid (C22:5n-6); and lower proportions of eicosapentaenoic acid (C20:5n-3) (*P*< 0.05). In all subjects, the NAFLD score was positively correlated with body mass index (BMI) (kg/m^2^) (r = 0.696), homeostasis model of assessment ratio–index (HOMA-IR) (r = 0.510), SCD1_(16)_ (r = 0.273), and the delta-6 index (r = 0.494); and inversely associated with the delta-5 index (r = -0.443).

**Conclusion:**

Our current data suggested that children with liver steatosis was highly associated with obesity, and insulin resistance. In addition, increased endogenous lipogenesis through altered desaturase activity may contribute to the progression of liver steatosis in children.

## Introduction

Concomitant with the rise of obesity, nonalcoholic fatty liver disease (NAFLD) has become the most common cause of chronic liver disease in children and adolescent [1–3]. Children with NAFLD are associated with insulin resistant, hypertension and dyslipidemia, which are collectively defined as the metabolic syndrome [4] and have an increased risk for cardiovascular disease and diabetes in later life [5–7]. Risk factors contributing to the development and progression of NAFLD have not been fully elucidated [2,3], particularly in children.

It is known that when the nutrient intake exceeds expenditure, tissues such as the adipose, liver, and skeletal tissues become saturated with lipids resulting in an increase in lipid export and leading to elevated plasma levels of free fatty acids (FAs) [8, 9]. Previous studies have shown that obese children had significantly higher concentrations of circulating fatty acids compared with normal-weight controls [10]. Studies indicated that FAs composition in blood was correlated with the FAs composition in liver [11]. Research on adult subjects demonstrated that both hepatic and serum FA compositions differed in non-alcoholic steatohepatitis patients, subjects with simple liver steatosis, and healthy controls [11, 12]. In addition, disease severity is directly related to the level of circulating FAs [13]. Furthermore, Docosahexaenoic acid supplementation has been experienced as potential treatment for pediatric NAFLD [14]. Taken together, all these findings suggest a vital role of FAs in the development of NAFLD and FAs may associate with the severity of liver injury.

In human studies, the use of plasma desaturase activity as a surrogate marker reflecting liver enzyme activity is well established [15, 16]. Among desaturase indices, stearoyl-coenzyme A desaturase-1 (SCD1) is a key enzyme in fatty acid and energy metabolism [17]. Increased hepatic SCD1 activity is associated with obesity and obesity-related diseases [17], SCD1_(18)_ converts the saturated FA, stearic acid (18:0), into the monounsaturated FA, oleic acid (18:1n-9). SCD1_(16)_ converts palmitic acid (16:0) into palmitoleic acid (16:1n–7). Furthermore, Δ5 and Δ6 desaturases are responsible for the processing of long-chain polyunsaturated FAs [18], which are estimated as the product/ precursor ratio of delta-5 (Δ5) desaturase = (20:4 n-6 / 20:3n-6) and delta-6 (Δ6) desaturases = (20:3n-6 /18:2n-6).

To date, few studies have investigated the FA composition pertaining to NAFLD in children [14, 19]. In the present study, we first compared anthropometric, biochemical data and concentrations of plasma FAs between children with liver steatosis and healthy controls. Second, we investigated FA composition and the association of FA composition among children with high-grade liver steatosis, low-grade liver steatosis, and healthy controls. Third, we analyzed the activity of desaturase indicators (SCD1, Δ5 and Δ6 desaturases) that are involved in FA synthesis and degradation, and unraveled the roles of these indicators in the pathogenesis of NAFLD.

## Methods

### Study subjects

In this study, we recruited obese and overweight children aged 8–18 years, who were willing to receive obesity measure and fatty liver screening from outpatient clinics of the Chang Gung Memorial Hospital, Keelung, Taiwan and healthy controls aged 13–18 years from the **P**rediction of **A**llergies in **T**aiwanese **CH**ildren (PATCH) cohort study [20] from March 2015 to August 2016. The parents and children received both verbal and written information regarding the aims and design of our study. All the participants were enrolled after obtaining written informed consent from their parents. Demographic data were obtained from participants and their parents through questionnaires. Subjects who received medications that could affect glucose metabolism, or who were diagnosed with chronic hepatitis, including hepatitis B, hepatitis C, autoimmune hepatitis, and Wilson’s disease were excluded from the study. This study was approved by the Ethics Committee of Chang Gung Memory Hospital (104-7100C), and was in line with the declaration of Helsinki.

### Anthropometric evaluation

Anthropometric measurements (height, weight, and waist and hip circumferences) of the children were taken. Height and weight were used to calculate body mass index (BMI) in kg/m^2^. Children with BMI ≥ 95th percentile were defined as obese, with BMI ≥ 85-95th percentile as overweight, and with BMI ≥ 10th-85th percentile as normal weight. Age- and gender-adjustments were performed according to the standard of the Department of Health in Taiwan [21]. Waist circumference was measured at the level of the umbilicus. Hip circumference was measured at the point of maximal protrusion of the buttocks. Waist-to-hip and waist-to height ratios were calculated from these measurements.

### Liver ultrasonographic examinations and scoring system for liver steatosis

Assessment of liver steatosis was carried out using a high-resolution B-mode scanner (Philips iE 33) machine. Liver ultrasound examinations and scoring for liver steatosis were performed by the same pediatric gastroenterologist (10-year experience). The ultrasonography rating of liver steatosis in this study included three scoring items that have been described by Hamaguchi M. et al. [22]. This score is referred to as the NAFLD score in this study and is composed of: (1) Bright liver and hepatorenal echo contrast (score 0–3); (2) deep attenuation of diaphragm (score 0–2); (3) visualization of intrahepatic vessels (score 0–1) [22]. Standardized views of the liver were obtained to enable scoring of these three items (NAFLD score 0–6). If the score for the hepatorenal echo contrast and bright liver was ≥ 1, we summed up all scores and defined the subjects as having liver steatosis. If hepatorenal echo contrast and bright liver scores were zero, the total score was defined as zero.

### Biochemical analysis

Blood samples of 10 ml were collected in tubes containing 1 g EDTA/l after subjects had fasted for at least 12 h. Plasma was separated within 3 h by centrifugation (3000 rpm for 10 min) at room temperature. Blood samples were analyzed for alanine aminotransferase (ALT), asparte aminotransferase (AST), gamma glutamyl transferase (γ-GT), glucose, insulin, triglyceride, low density lipoprotein-cholesterol (LDL-C), and high density lipoprotein -cholesterol (HDL-C). HOMA-IR (homeostasis model of assessment ratio–index) was also obtained by using Matthews et al’s formula as an index of insulin resistance [23]. Hepatitis B and C, autoimmune hepatitis, and Wilson disease were excluded using the appropriate diagnostic tests.

### Plasma fatty acids analysis

Plasma FA methyl esters were prepared as described previously (Moser et al., 1999) [24]. Briefly, 200 μl plasma and 10 μg C13:0 as an internal standard were mixed with 1 ml methanol/methylene chloride 3:1 (v/v). Subsequently, 200 μl of acetyl chloride, were added and the samples were placed in a 75°C water bath for 1 h. After cooling down, 4 ml of 7% potassium carbonate and 2 ml hexane were added, and samples were mixed and centrifuged for 10 min at 3000 rpm at room temperature. The plasma sample in hexane layer was dried using nitrogen gas and analyzed with Agilent 7820A GC using flame ionization detection on a SP-2560 polar fused silica capillary column (100 m x 0.25 mm x 0.2 μm, Supelco Inc.) with nitrogen as carrier gas. The oven temperature program was initially set to 60°C for 1 min, then increased by 25°C per minute to 160°C, then increased by 2°C per minute to 240°C for 10 min, and finally increased by 5°C per minute to 245°C for 5 min. The FA peaks were identified by comparing retention times from our samples with retention times of a standard mixture of GLC-68A, GLC-481, GLC-532, GLC-744 (Nu-Chek Prep), 37 FAME, cis/trans 18:2n-6 and cis/trans 18:3n-3, (all from SUPELCO). The FA composition was expressed as the weight of a percentage of the total weight of carbon 12 to carbon 24 FAs (wt%). http://dx.doi.org/10.17504/protocols.io.h6hb9b6

### Definitions used in this study

#### Liver steatosis group

Obese (BMI ≥ 95th percentile) or overweight (BMI ≥ 85–95 percentile) children with sum ultrasound scores for liver steatosis (NAFLD score) ≥ 1, negative for chronic hepatitis including hepatitis B, hepatitis C, autoimmune hepatitis, and Wilson’s disease. In this study, sum ultrasound scores for liver steatosis (NAFLD score) of 4–6 were defined as ***high-grade liver steatosis*,** and NAFLD score 1–3 were defined as ***low-grade liver steatosis*.**

#### Healthy controls group

Children with normal weight (BMI ≥ 10-85th percentile), sum ultrasound scores for liver steatosis zero (NAFLD score = 0), and no medical disorders.

#### Estimated desaturase indices

SCD1_(16)_ was calculated as the ratio of 16:1n–7 to 16:0; SCD1_(18)_ as the ratio of 18:1n–9 to 18:0; Δ5 desaturase as the ratio of C20:4n-6 to C20: 3n-6; and Δ 6 desaturase as the ratio of C20:3n-6 to C18: 2n-6 [17, 18].

### Statistical analysis

Categorical variables were compared using the χ 2 test, continuous variables were expressed as mean ± SD and were analyzed by using Student's two-tailed t test or a one-way ANOVA analysis variance. Correlation analyses were performed by using Pearson’s correlation analysis for variables normally distributed and by Spearman’s test for skewed variables. We considered P< 0.05 as statistically significant. Statistical analysis was performed using IBM SPSS statistics version 20 (Armonk, NY, USA).

## Results

A total of 130 schoolchildren (aged 8–18 years) were recruited. Each subject completed anthropometric measurements, liver ultrasound examination and scoring for liver steatosis (NAFLD score), and biochemical and plasma FA analysis. To avoid potential confounding factors, 10 obese or overweight children who had no ultrasound liver steatosis and 9 normal-weight children who had ultrasound liver steatosis were excluded. Our final sample included 111 schoolchildren.

The characteristics of the subjects participating in this study are summarized in Table 1. Among our subjects, 51 (45.9%) were obese, 8 (7.2%) were overweight, and 52 (46.8%) were normal-weight and were considered as healthy controls. BMI (kg/m^2^), waist-hip and waist-height ratio were significantly higher in the liver steatosis group than in the control group. There was no difference between the two groups in terms of mean age, and gender. When analyzing the biochemical data, we found that Insulin, HOMA-IR-index, ALT, AST, γ-GT, Triglyceride levels were significantly higher, and HDL-C levels were significantly lower in liver steatosis children than controls. The serum total cholesterol and LDL-C did not differ significantly between 2 groups (Table 1).

**Table 1:** Comparison of demographic, biochemistry, and fatty acids data in children with liver steatosis and healthy controls.

	Healthy controls (n = 52)	Children with liver steatosis (n = 59)	P value
**Basic and anthropometric data**			
Age (y)	15.28 ± 1.80	14.13 ± 3.63	0.101
Gender, male (%)	23 (44.2)	35 (59.3)	0.088
BMI (kg/m2)	19.10 ± 1.66	27.56 ± 2.94	<0.001*
Waist-hip ratio	0.77 ± 0.05	0.89 ± 0.09	<0.001*
Waist-height ratio	0.42 ± 0.04	0.57 ± 0.08	<0.001*
**Biochemical data**			
Fasting blood glucose (mg/dL)	87.82 ± 5.17	89.51± 7.34	0.399
Insulin (μlU/ml)	4.76 ± 3.80	12.05 ± 8.06	<0.001*
HOMA-IR	1.36 ± 0.67	3.33 ± 2.33	0.008*
ALT (U/L)	13.08 ± 6.23	40.13 ± 45.24	<0.001*
AST (U/L)	17.81 ± 7.34	29.65 ± 22.28	0.001*
γ-GT (U/L)	13.63 ± 5.49	20.66 ± 10.69	<0.001*
Triglycerides (mg/dL)	74.87 ± 72.38	115.67± 62.31	0.010*
Total cholesterol (mg/dL)	166.27 ± 35.75	174.08 ± 27.85	0.273
HDL-C (mg/dL)	52.67± 14.69	42.85 ± 6.94	0.010*
LDL-C (mg/dL)	92.42 ± 26.44	103.00 ± 24.38	0.272
**Plasma fatty acids**			
SFA (μg/ml)	859.93 ± 219.53	1045.70 ± 306.96	<0.001*
MUFA(μg/ml)	491.43 ± 127.64	637.38 ± 228.18	<0.001*
n6-PUFA(μg/ml)	1042.42 ± 213.49	1209.46 ± 284.79	0.001*
n3-PUFA(μg/ml)	94.18 ± 32.28	121.17 ± 38.93	<0.001*
**Desaturase index**			
SCD1_(16)_	0.06 ± 0.02	0.07 ± 0.02	0.038*
SCD1_(18)_	1.82 ± 0.52	1.95± 0.45	0.180
Δ5	6.07 ± 1.60	4.71 ± 1.63	<0.001*
Δ6	0.03 ± 0.01	0.05± 0.01	<0.001*

*Significance levels: *P*< 0.05

### Plasma fatty acids composition in children with liver steatosis

In general, children with liver steatosis according to our criteria had significant higher levels of total plasma FAs (3013.71 ± 795.03 μg/ml *vs*. 2487.96 ± 499.95 μg/ml, *p* < 0.001), and saturated FA (SFA), monounsaturated FA (MUFA), n6-polyunsaturated FA (PUFA), and n3-PUFA levels than healthy controls *(p* ≤ 0.001, Table 1). Analysis of FA composition showed that children with liver steatosis exhibited a higher proportion of MUFAs than healthy controls (21.16 ± 2.81% *vs*. 19.68 ± 2.71%, *p* = 0.024). However, proportions of saturated FAs (34.58 ± 3.19% *vs*. 34.51 ± 4.43%, *p* = 0.922), n6-PUFA (40.52 ± 4.00% *vs*. 42.05 ± 4.52%, *p* = 0.059), and n3-PUFA (4.05 ± 0.97% *vs*. 3.76 ± 0.89%, *p* = 0.103) were not significantly different in the two groups.

Next, we analyzed plasma FA profiles in children with high-grade, low-grade liver steatosis and healthy controls. Children with high-grade liver steatosis showed a higher proportion of palmitic acid (C16:0), palmitoleic acid (C16:1n-7), dihomo-γ-linolenic acid (C20:3n-6), adrenic acid (C22:4n-6), and docosapentaenoic acid (C22:5n-6); and a smaller proportion of eicosapentaenoic acid (C20:5n-3) than children with low-grade liver steatosis and healthy controls (*p* < 0.05, Table 2). In addition, Δ5, and Δ 6 desaturase activity levels were significantly different among the three groups (*p* < 0.05, Table 2).

**Table 2:** Compositions and proportions of plasma fatty acids in children with high- and low-grade liver steatosis and healthy controls.

	Healthy controls (n = 52)	Children with low-grade liver steatosis (n = 27)		Children with high-grade liver steatosis (n = 32)
	Mean (%) ± SD	Mean (%) ± SD		Mean (%) ± SD
**SFA**	34.51 ± 4.43	34.22 ± 4.26		34.88 ± 1.89
C14:0	0.52 ± 0.23^a^	0.53 ± 0.17^ab^		0.62 ± 0.22^b^
C16:0	22.85 ± 1.74^a^	22.70 ± 1.76^a^		23.66 ± 1.25^b^
C17:0	0.24 ± 0.09^a^	0.22 ± 0.08^ab^		0.19 ± 0.05^b^
C18:0	9.01 ± 2.82	8.94 ± 2.52		8.61 ± 0.97
C20:0	0.32 ± 0.08	0.31± 0.09		0.28 ± 0.08
C22:0	0.91 ± 0.23	0.87 ± 0.19		0.87 ± 0.27
C24:0	0.67 ± 0.26	0.66 ± 0.28		0.64 ± 0.21
**MUFA**	19.68± 2.71^a^	20.28± 2.53^ab^		21.34 ± 2.83^b^
C14:1	0.12 ± 0.05^a^	0.11 ± 0.05^ab^		0.09 ± 0.04^b^
C16:1n-7	1.30± 0.45^a^	1.35± 0.40^a^		1.70± 0.56^b^
C18:1n-9	15.30 ± 2.29^a^	16.03 ± 2.34^ab^		16.87 ± 2.46^b^
C18:1n-7	1.39 ± 0.21^a^	1.33 ± 0.16^ab^		1.26 ± 0.20^b^
C20:1n-9	0.10 ± 0.07	0.10 ± 0.05		0.09 ± 0.05
C24:1n-9	1.48 ± 0.41	1.37 ± 0.38		1.31 ± 0.48
**n6-PUFA**	42.05± 4.52^a^	41.27± 4.18^ab^		39.88 ± 3.79^b^
C18: 2n-6	34.37 ± 4.44^a^	32.94 ± 4.23^ab^		31.32 ± 3.43^b^
C18:3n-6	0.20± 0.20^a^	0.25± 0.26^ab^		0.31 ± 0.20^b^
C20:3n-6	1.06 ± 0.30^a^	1.30 ± 0.20^b^		1.54 ± 0.25^c^
C20:4n-6	6.10± 1.21	6.45 ± 1.42		6.31 ± 1.03
C22:4n-6	0.19 ± 0.07^a^	0.19 ± 0.05^a^		0.22 ± 0.06^b^
C22:5n-6	0.13± 0.04^a^	0.15± 0.05^a^		0.18 ± 0.05^b^
**n3-PUFA**	3.76 ± 0.89^a^	4.22 ± 1.24^b^	3.90 ± 0.64^ab^	
C18:3n-3	0.51± 0.15^a^	0.59± 0.19^ab^	0.61 ± 0.20^b^	
C20:5n-3	0.68 ± 0.30^ab^	0.74 ± 0.43^a^	0.56 ± 0.23^b^	
C22: 5n-3	0.36 ± 0.09^a^	0.40 ± 0.09^b^	0.40± 0.10^ab^	
C22:6n-3	2.21 ± 0.62	2.50 ± 0.81	2.33 ± 0.54	
**Desaturase index**				
SCD1_(16)_	0.06 ± 0.02^a^	0.06 ± 0.02^a^	0.07 ± 0.02^b^	
SCD1_(18)_	1.82 ± 0.52	1.89 ± 0.49	1.99 ± 0.41	
Δ5	6.07 ± 1.60^a^	5.32 ± 2.08^b^	4.19 ± 0.85^c^	
Δ6	0.03 ± 0.01^a^	0.04 ± 0.01^b^	0.05 ± 0.01^c^	
**Plasma fatty acids**				
Total fatty acids (μg/ml)	2487.96 ± 499.95^a^	2848.30 ±599.27^b^	3153.27 ± 915.13^b^	

Different letters in the superscripts showed significant differences between groups (*P* < 0.05)

### Associations between metabolic variables, NAFLD score and fatty acids

Correlation coefficients (r) are summarized in Table 3. The grading of liver steatosis was strongly associated with BMI and insulin resistance. We observed a stronger correlation between BMI (kg/m^2^) and NAFLD scores (r = 0.696, *p* < 0.001) than HOMA-IR and NAFLD scores (r = 0.510, *p* < 0.001). Except for palmitic acid (C16:0) and γ-linoleic acid (C18:3n-6), the correlations between BMI (kg/m^2^) and NAFLD scores for individual FAs and estimated desaturase ratios followed a similar pattern. Three variables, BMI, HOMA-IR and NAFLD score, all showed positive correlations with palmitoleic acid (C16:1n-7), oleic acid (C18:1n-9), dihomo-γ-linolenic acid (C20:3n-6), SCD1_(16)_ (C16:1n-7 /C16:0), and the Δ6 (C20:3n-6/C18: 2n-6) index, and negative correlations with linoleic acid (C18: 2n-6), and the Δ5 (C20:4n-6 /C20: 3n-6) index.

**Table 3:** Associations (r) between the relative content of plasma fatty acids (% of total fatty acids), desaturase activity and selected factors for liver steatosis.

	HOMA-IR	BMI (kg/m2)	NAFLD score (0–6)
**BMI (kg/m2)**	0.365*	-	0.696***
**NAFLD score (0–6)**	0.510***	0.696***	-
**C16:0**	0.514***	NS	0.208*
**C16:1n-7**	0.386*	0.258**	0.318***
**C18:0**	NS	NS	NS
**C18:1n-9**	0.431**	0.210*	0.273**
**C18: 2n-6**	-0.353*	-0.268**	-0.275**
**C18: 3n-6**	NS	NS	0.172*
**C20:3n-6**	0.307*	0.510***	0.515***
**C20:4n-6**	-0.354*	NS	NS
**SCD1_(16)_**	0.306*	0.231**	0.273**
**SCD1_(18)_**	NS	NS	NS
**Δ5**	-0.434**	-0.408***	-0.443***
**Δ6**	0.378*	0.485***	0.494***

Significance levels: *P*< 0.05*, P< 0.01**, P<0.001 ***, NS: P>0.05

## Discussion

In this study, we demonstrated associated risk factors and differences in FA proportions and desaturase activities among obese/overweight children with different grades of liver steatosis. It is known that obesity and insulin resistance are the main risk factors for pediatric NAFLD [25]. Overweight and obese children have been reported to account for 81% of all cases of fatty liver disease [26], and insulin resistance would result in increased lipolysis from adipose tissue and increased lipid influx into the liver, promoting hepatic fat accumulation [27–29]. Consistently, our results indicate that both higher BMI and insulin resistance increase the likelihood of severe liver steatosis. Furthermore, we found that BMI (kg/m^2^) is more impactful for child liver steatosis (r = 0.696, *p* < 0.001) than insulin resistance (r = 0.510, *p* < 0.001, Table 3).

It was considered that imbalance between fatty acid uptake and disposal, increased de novo hepatic lipogenesis, and high dietary lipid intake were involved in the pathogenesis of NAFLD [27, 29, 30], but have not been fully investigated in children. To assess the role of fatty acids in pediatric NAFLD, we first compared the FAs compositions among children with liver steatosis, and healthy controls. We found concentrations of total plasma FAs and proportions of MUFAs were significantly elevated in obese and overweight children [7, 8, 10] with liver steatosis as compared with healthy controls (Table 1). Similarly, previous studies have mentioned that MUFA is correlated with hepatic steatosis [29] and altered MUFA composition may play a role in the development of metabolic syndrome [7]. Second, we found there were multiple differences in plasma FA proportions between children with high- and low-grade liver steatosis (Table 2). Among those plasma FAs, palmitoleic acid (C16:1n-7), and dihomo-γ- linolenic acid (C20:3n-6) has positive correlation with NAFLD score, as well as BMI and HOMA-IR (Table 3). In literatures the question whether elevated plasma palmitoleic acid (C16:1n-7) has adverse effects on insulin resistance and metabolic disorder remains controversial. In animal and in vitro studies, palmitoleic acid (C16:1n-7) has been found to facilitate uptake and utilization of glucose in normal and insulin-resistant skeletal muscles, and to suppress hepatosteatosis [31, 32]. These results indicate potential benefits of palmitoleic acid on metabolic disorders. However, several human studies and our findings seem to contradict these results [7, 33, 34]. Our results suggest that increased concentrations of palmitoleic acid (C16:1n-7) and dihomo-γ- linolenic acid (C20:3n-6) contribute to unfavorable metabolic outcomes [7, 33, 34], and are associated with hepatic lipogenesis [35].

It is notable that differences in diets would influence the levels of some fatty acids in blood. Serum FA profile such as linoleic acid (18:2n-6) and alpha linolenic acid (18:3n-3) are not synthesized by humans and reflect dietary intake of the last two to six weeks [36]. In contrast, palmitoleic acid (16:1n -7) and dihomo-γ-linolenic acid (C20:3n-6) are less dependent on intake and could reflect endogenous lipogenesis [7, 15]. In the present study, the explanations for the elevated palmitoleic acid (16:1n-7) and dihomo-γ-linolenic acid (C20:3n-6) levels in our subjects with high-grade liver steatosis probably resulted from the change of desaturase activity, including higher expression of SCD1_(16)_ (C16:1n-7/C16:0), higher Δ6 activity (C20:3n-6 /C18: 2n-6), and lower Δ5 activity (C20:4n-6/C20: 3n-6), accelerating endogenous lipogenesis. In agreement with studies in adults, we observed that changes of these desaturase indices were significantly correlated to NAFLD score in children (Table 3), which implies that desaturase activity plays a role in progression of liver steatosis [11, 15, 35, 37]. Although the detailed information about dietary intake was lacking, the proportions of essential fatty acids such as 18:2n -6, and 18:3n-3 showed no difference between children with high and low-grade liver steatosis (Table 2), probably not associated with the severity of liver steatosis. On the basis of the above-mentioned information, we suggested that change in SCD1, Δ5 and Δ6 desaturase activities, and acceleration of endogenous lipogenesis are key determinants to the progression of liver steatosis in children.

Recently, studies reported a relationship between lower n-3 PUFA levels and NAFLD [38–40], and supplementation with eicosapentaenoic acid (C20:5n-3) and docosahexaenoic acid (C22:6n-3) have potential benefits for the prevention and treatment of NAFLD [14, 19, 38, 41]. Although the proportion of eicosapentaenoic acid (C20:5n-3) was significant lower in the high-grade liver steatosis group than in the low-grade liver steatosis group (Table 2), the present study did not show a downward trend in the proportions of eicosapentaenoic acid (C20:5n-3) and docosahexaenoic acid (C22:6n-3) in the subjects with liver steatosis as compared with the healthy controls, which is contrary to previous publications. A possible explanation for the contradicted result is that the dietary intakes regarding eicosapentaenoic acid (C20:5n-3) and docosahexaenoic acid (C22:6n-3) did not differ among the subjects with liver steatosis and the healthy controls, no significant influence to NAFLD. As all the participants lived in Keelung, which is home to the major seaport in northern Taiwan, consuming fish is easy.

The significance of the study was the young age of our participants (mean age, 14.87 ± 2.55 years). Although several studies in adults have documented changes of desaturase activity and increased synthesis of MUFA in obesity and NAFLD conditions [11, 15, 35, 37], to date, only few studies in children have addressed this issue [19]. Whether pediatric NAFLD presents similar pathogenesis as adult NAFLD remains uncertain. In addition, this is the first study to demonstrate the differences in FA proportions and desaturase activities between children with different liver steatosis ratings. The present study has some limitations. One major limitation is the diagnostic modality used in this study. Rather than using liver biopsies, children were diagnosed based on ultrasonography. Although a number of studies demonstrated that ultrasonography shows relatively high sensitivity (82–94%) and specificity (66–95%) in detecting fatty liver [42, 43], liver ultrasonography has been considered to be an imperfect screening tool for NAFLD. Therefore, in the current study, we applied a scoring system for the evaluation of fatty liver on the basis of three ultrasonographic parameters: hepatorenal echo contrast and liver brightness, deep attenuation of diaphragm, and intrahepatic vascular blurring [22]. Hamaguchi M et al. reported that this scoring system provides accurate information about liver steatosis; the sensitivity and specificity of diagnosis reached 91.2–92.6% and 100%, respectively [22]. Another major limitation of our study is that we did not evaluate the effect of dietary intake. Reviewed literatures, some animal and human studies showed that SCD activity could be regulated by diet, which means that the dietary factor is important to the lipogenesis and development of NAFLD [44–46]. By contrast, Donnelly et al. described that the dietary lipid was not the major contributing factor to hepatic fat build-up (approximately 15%) as compared with elevated levels of peripheral fatty acids and de novo lipogenesis [30]. In addition, a diet-controlled study in Finland demonstrated that individuals with NASH had significant alterations in FA metabolism and endogenous desaturase activities, independent of obesity and diet [37]. Although our main findings of changes in fatty acid levels do not seem to be indicative of oral intake, whether dietary fatty acid intakes partly influenced our results remains to be elucidated. Thus, our data should be interpreted with caution. Finally, as this was a cross-sectional study, whether a change in FA composition in blood precedes liver steatosis or liver steatosis results in alternations of blood fatty acid profiles could not be known. Larger studies with a prospective design and detailed dietary assessment are needed to confirm our findings.

## Conclusions

Liver steatosis in children is strongly associated with obesity, and insulin resistance. In addition, we found that increased endogenous lipogenesis through altered desaturase activity may play a role in the progression of liver steatosis in children.

## Supporting information

S1 File Compositions and proportions of plasma fatty acids and NAFLD scores in our study subjects. (XLS)
